# Effect of divergences about patient's care plan on the outcome of critically ill patients

**DOI:** 10.1186/cc13210

**Published:** 2014-03-17

**Authors:** EL Queiroz, G Schettino

**Affiliations:** 1 Hospital Sírio-Libanês, Sao Paulo, Brazil

## Introduction

There is an actual discussion about the best way to provide medical care for critically ill patients, particularly between the classical opened versus closed ICU models [1,2]. In Brazil, all ICUs have to have a full-time and dedicated physician at the unit but the primary physician also can visit his patient every day and, most of the time, participates in the patient's care plan. This hybrid model creates opportunity for patient's care plan divergences between the ICU staff and the primary physician. The objective of this study is to evaluate the effect of divergences about patient's care plan on the outcome of critically ill patients.

## Methods

A prospective court study was conducted (from January to May 2013) to point out the patient's care divergences (blood transfusion, diuretics, antibiotics, vasopressors, mechanical ventilation, and so forth) that happened in the first 72 hours of ICU admission in a 30-bed adult Brazilian ICU. We enrolled only patients that stayed more than 48 hours in the ICU.

## Results

In a court of 357 patients at least one divergence between the ICU medical staff and the primary(s) physician(s) were identified in 31 cases (8.6% - divergence group (DG)). The age (67.9 years), gender (55.2% of male), SAPS3 score (45.7) and reasons for ICU admission (emergency surgery 7.6%, nonemergency surgery 30%, clinical 62.4%) were similar in DG and nondivergence (NDG); however, the ICU length of stay (6.2 vs. 3.9 days, *P *= 0.023), use of mechanical ventilation (48.4% vs. 27%, *P *= 0.012), vasopressors (77.4% vs. 46%, *P *= 0.001) and blood transfusion (41.9% vs. 27.6%, *P *= 0.073) were higher in the DG compared with NDG. Discordance was associated with higher ICU and hospital mortality (35.5 vs. 11%; OR = 4.09; *P *< 0.001 and 45.2 vs. 20.1%; OR = 2.77; *P *= 0.02 respectively).

## Conclusion

The occurrence of divergences, even during the first days of ICU admission, about medical plans of care for critically ill patients is frequent and is associated with higher ICU and hospital mortality and more use of medical resources.
